# Primary adrenal insufficiency and systemic tuberculosis in a 10-year-old boy: case report

**DOI:** 10.17843/rpmesp.2025.421.14092

**Published:** 2025-03-12

**Authors:** Sandra Schult-Montoya, Felipe Ricardo Lindo-Pérez

**Affiliations:** 1 Instituto Nacional de Salud del Niño, Lima, Peru. Instituto Nacional de Salud del Niño Lima Peru; 2 Universidad Nacional Mayor de San Marcos, Lima, Peru. Universidad Nacional Mayor de San Marcos Universidad Nacional Mayor de San Marcos Lima Peru

**Keywords:** Primary adrenal insufficiency, Addison’s disease, multisystemic TB, tuberculous adrenalitis, pediatrics

## Abstract

Primary adrenal insufficiency is the result of impaired steroid synthesis, adrenal destruction or abnormal development of the adrenal gland affecting the adrenal cortex. Tuberculosis is one of the main causes in developing countries. We present the case of a 10-year-old male patient diagnosed with adrenal insufficiency two years earlier with regular treatment, who was admitted for adrenal crisis. A thoraco-abdominal tomography was carried out during hospitalization, which showed cylindrical and traction bronchiectasis associated with cicatricial atelectasis in the right upper pulmonary lobe, enlarged adrenal glands with foci of calcification, as well as a dense posterior perivertebral mediastinal collection also involving vertebral bodies with lytic resorptive foci, findings consistent with systemic tuberculosis. Treatment for tuberculosis started based on this diagnosis. During course of the disease, the first phase was completed with four drugs, and the second phase included isoniazid and rifampicin with favorable clinical response.

## INTRODUCTION

Primary adrenal insufficiency (AI), also known as Addison’s disease (AD), is caused by a condition intrinsic to the adrenal cortex. This condition results from impaired steroid synthesis, adrenal destruction, or abnormal development of the gland affecting the adrenal cortex ^1^.

In children, the etiology varies with age. In newborns and infants, the most important cause is congenital, such as congenital adrenal hyperplasia due to 21-hydroxylase enzyme deficiency. In older children, it may be due to acquired causes such as autoimmune causes, infiltrative processes, or infections; tuberculosis being one of the latter ^2^.

The distribution of specific causes of primary AI varies according to the population ^3^^-^^5^. Overall, the most common causes in male children in developed countries are autoimmune and adrenoleukodystrophy, which account for 80-90% of all cases ^6^^-^^8^; whereas in developing countries, tuberculosis is still one of the main causes ^9^^,^^10^.

We present the case of a patient diagnosed with primary AI who was admitted for adrenal crisis and diagnosed with systemic tuberculosis during evaluation.

## CASE REPORT

**Figure 1:**
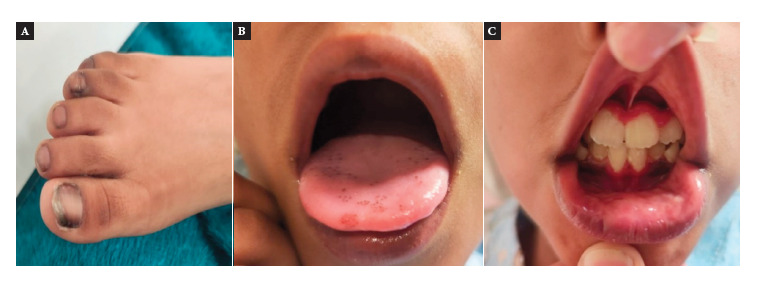
Skin hyperpigmentation.

A 10-year-old male patient with history of adrenal insufficiency diagnosed two years prior to admission. He was monitored as an outpatient and received regular treatment with hydrocortisone and fludrocortisone. Two weeks prior to admission, he presented diffuse, moderate abdominal pain associated with a feeling of fever (occurring every other day). One week prior, he developed pain in the dorsal region and profuse sweating, mainly at night. Three days prior to admission, his symptoms worsened, with the addition of nausea and weakness when walking, leading to his admission to the emergency department. Physical examination revealed a heart rate of 107 beats per minute, respiratory rate of 24 breaths per minute, temperature of 36.7 °C, weight of 23 kilograms, height of 137 cm, blood pressure of 79/54 mmHg (below the 5th percentile for age and height), BMI of 12.25 (below the 1st percentile), capillary refill time of less than 2 seconds, spontaneous breathing, and signs of dehydration. Skin hyperpigmentation was also evident, predominantly on the fingers and toes, nails (Figure 1A) and genitals, with hyperchromic spots on the tongue and gums (Figure 1B, 1C). The abdomen was soft, depressible, and diffusely painful on deep palpation, with no visceromegaly. The rest of the physical examination was unremarkable.

On admission, hemoglobin was 11.6 g/dL, leukocytes 12,260/uL, platelets 660,000/uL, glucose 110 mg/dL, CRP 17.69 mg/dL, urea 71 mg/dL, creatinine 1.28 mg/dL, sodium 129 mmol/L, potassium 5.2 mmol/L, and chloride 96 mmol/L. Blood gas analysis showed the following result, pH: 7.36, pO_2_: 46, pCO_2_: 26.4, HCO_3_: 15.1. As well as, cortisol (AM): 5.9 ug/dL, ACTH: 50.2 pg/mL, TSH: 7.18 uUI/ml, free T4: 20.1 pmol/L; anti-thyroglobulin and anti-thyroid peroxidase antibodies were negative. The coagulation profile was as follows, prothrombin time (PT): 14.8 seconds, INR: 1.26, activated partial thromboplastin time (APTT): 37.4 seconds, thrombin time (TT): 15.7 seconds, fibrinogen: 702 mg/dL, and urine test: pH 6.0, specific gravity: 1020, clear appearance, ketones 2+, glucose and protein (-), leukocytes 1-2/field, red blood cells 1-2/field.

**Figure 2:**
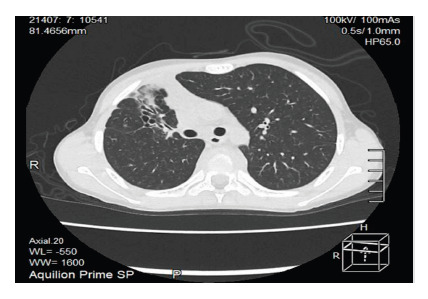
Cylindrical bronchiectasis in segment S3 on non-contrast chest tomography.

**Figure 3:**
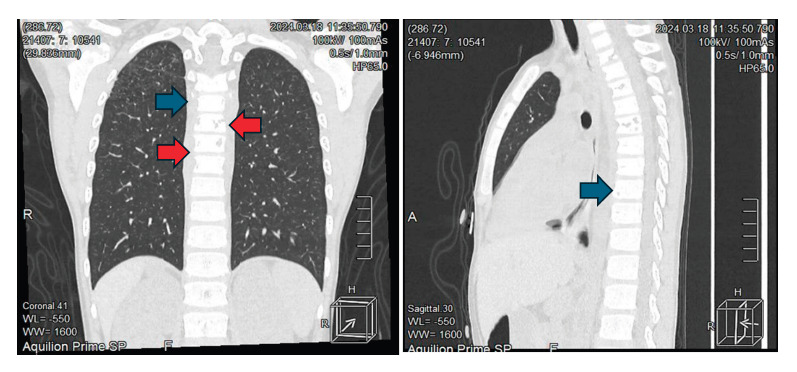
Residual lytic foci from T2 to T9 (blue arrow) and dense perivertebral mediastinal collection (red arrow) on non-contrast thoracoabdominal tomography.

**Figure 4:**
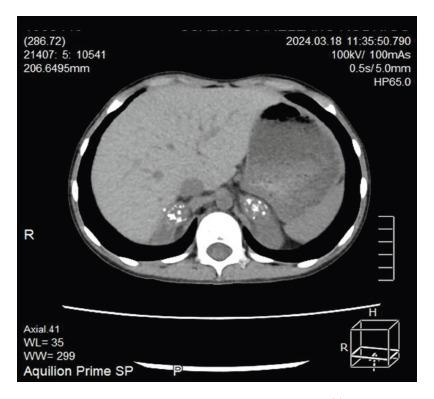
Bilateral adrenal calcifications on abdominal CT scan without contrast.

He was diagnosed with adrenal crisis and febrile syndrome and was prescribed a bolus of 0.9% NaCl 20 ml/kg on two occasions and intravenous hydrocortisone 100 mg/2ml. He was then kept hydrated with isotonic glucose-saline solution without potassium at a volume determined by the caloric method. BK and Gene-Xpert tests on gastric aspirate were negative. Chest X-ray showed atelectasis in the upper third of the right hemithorax. Abdominal ultrasound revealed enlarged adrenal glands with multiple calcifications, while the terminal ileum showed thickened walls with marked folds and thickening. Thoracoabdominal tomography showed cylindrical and traction bronchiectasis in the lungs associated with cicatricial atelectasis with fine micronodular infiltrates in the posterior segment S3 of the right upper lobe (Figure 2), as well as mediastinal lymphadenopathy, right paratracheal, infracarinal up to 15x12 mm, and some axillary lymphadenopathy. In addition, there was evidence of dense posterior mediastinal peri-vertebral collection in most of the dorsal axis with anterior subligamentary extension (Figure 3, red arrows), which also involved vertebral bodies with resorptive lytic foci and osteopenic changes from the second to the ninth thoracic vertebra (Figure 3, blue arrows). Both adrenal glands showed a slight increase in volume, containing thick foci of calcification (Figure 4), and the spleen showed a punctiform focus of calcification.

**Figure 5:**
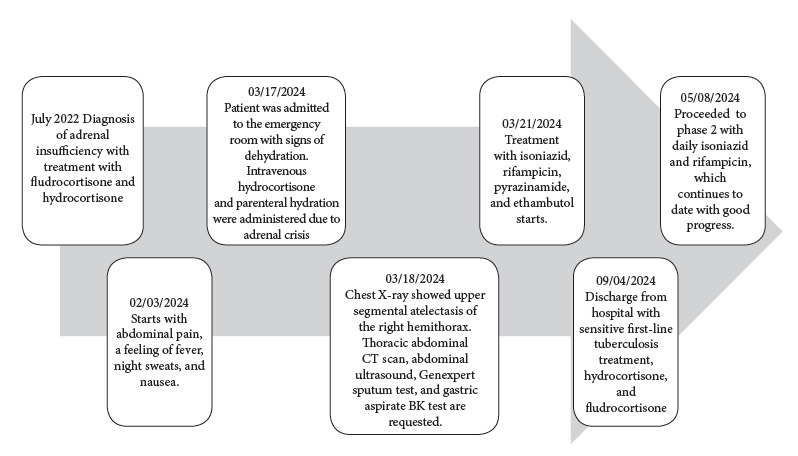
Timeline of clinical presentation and progression.

The diagnosis was multisystemic tuberculosis with pulmonary, vertebral, adrenal, and splenic involvement, therefore, antituberculosis treatment started with a sensitive regimen of isoniazid 10 mg/kg/day, rifampicin 15 mg/kg/day, etambutol 20 mg/kg/day, and pyrazinamide 30 mg/kg/day for four months in the first phase, followed by isoniazid and rifampicin daily in the second phase, which continues to the present day. Endocrinology discharged the patient with hydrocortisone 1 mg/kg/day and fludrocortisone 0.1 mg/day with favorable progress (Figure 5).

## DISCUSSION

When chronic adrenal failure was first described, around 50% of cases were secondary to tuberculosis. *Mycobacterium* tuberculosis causes invasion and destruction of the adrenal cortex, producing granulomas and necrosis, with subsequent fibrosis and calcification, leading to adrenal failure that may be irreversible ^11^. Subsequent reports show a decrease in the frequency of tuberculous adrenalitis as a cause of AD; however, in countries where tuberculosis is endemic, or in immunocompromised populations, this decrease is not as prominent, with an incidence rate ranging from 4.7 to 8.3 per million ^11^^,^^12^. Tuberculosis can primarily affect both adrenal glands or can be part of a disseminated disease, as in our case. Previous studies report that single adrenal gland involvement is not uncommon, but in most cases, other organs are also involved ^13^.

The clinical signs of AD are based on glucocorticoid and mineralocorticoid deficiency. Signs due to glucocorticoid deficiency include weakness, anorexia, and weight loss. Mineralocorticoid deficiency contributes to hyponatremia, hyperkalemia, acidosis, tachycardia, and hypotension ^1^. In our patient, hyponatremia and acidosis were evident at the time of diagnosis, as well as clinical hypotension requiring treatment with 0.9% NaCl boluses. The lack of negative feedback from glucocorticoids is responsible for the elevated ACTH levels at the time of the initial diagnosis of AI, with values still slightly high currently, despite the fact that treatment was not discontinued at any time. Elevated levels of ACTH and other melanocortin peptides (POMC), including several forms of melanocyte-stimulating hormone (MSH), cause hypersecretion of melanin, stimulating mucosal and cutaneous hyperpigmentation ^1^, which persists in the patient and intensifies during the crisis. Hyperpigmentation is usually a specific sign of AI and appears when there is more than 90% adrenal gland involvement as a result of the effect of increased ACTH and MSH ^14^.

Acute adrenal crisis is a potentially life-threatening condition in all age groups. Patients present profound discomfort, fatigue, nausea, vomiting, abdominal or side pain, muscle aches or cramps, and dehydration, leading to hypotension, shock, and metabolic acidosis ^1^. Our patient was found to have hypovolemic shock with nitrogen retention due to prerenal injury secondary to dehydration. This condition requires immediate treatment, which is based on rapid fluid replacement to correct hypovolemia, electrolyte imbalance, and hypoglycemia, as well as the administration of intravenous hydrocortisone ^(1, 2)^, as described in the present case.

The clinical description upon admission is typical of an adrenal crisis, despite having received regular replacement therapy. This highlights the importance of determining the etiology of adrenal insufficiency for adequate control. Due to the fever without an obvious cause, a chest X-ray was requested, followed by a chest and abdominal CT scan, which revealed pulmonary involvement, as well as involvement of the thoracic vertebrae, adrenal glands, and spleen.

Finding enlarged adrenal glands is reported in multiple studies of tuberculous adrenalitis ^15^, especially when the process of the disease is around 3 years ^16^. In this case, bilateral calcifications are evident on the CT scan, which is usually found as more time passes after the tuberculous infection. These findings, associated with asymptomatic vertebral involvement, are consistent with the chronic course of the disease.

Since tuberculous adrenalitis involves destruction of more than 80% of the adrenal gland before symptoms appear, antituberculosis treatment does not always result in complete recovery of adrenal function ^13^; however, better adrenal control has been reported after treatment starts. In our case, treatment started with a sensitive regimen that included isoniazid, rifampicin, ethambutol, and pyrazinamide, then treatment continued with hydrocortisone and fludrocortisone.

In countries such as Peru, with a high prevalence of tuberculosis, tuberculous adrenalitis should always be considered in the differential diagnosis of schoolchildren and adolescents presenting with primary AI.
