# Supplementary material for: Primary adrenal insufficiency and systemic tuberculosis in a 10-year-old boy: case report
Source: Rev Peru Med Exp Salud Publica. 2025 Mar 12;42(1):92–6. doi: 10.17843/rpmesp.2025.421.14092 (PMC12176017; doi:10.17843/rpmesp.2025.421.14092)
Supplement: Translation

# Translation: Insuficiencia suprarrenal primaria y tuberculosis sistémica en un varón de 10 años. Reporte de caso

**Authors:** Sandra Schult-Montoya, Felipe Ricardo Lindo-Pérez

**Affiliations:** 1 Instituto Nacional de Salud del Niño, Lima, Perú.; 2 Universidad Nacional Mayor de San Marcos, Lima, Perú.

**Keywords:** Insuficiencia suprarrenal primaria, Enfermedad de Addison, tuberculosis multisistémica, Adrenalitis tuberculosa, pediatría

## Abstract

La insuficiencia suprarrenal primaria es resultado de una síntesis alterada de esteroides, destrucción suprarrenal o desarrollo anormal de la glándula que afectan a la corteza suprarrenal. Siendo la tuberculosis una de las principales causas en países en vías de desarrollo. Presentamos el caso de paciente varón de 10 años con diagnóstico de insuficiencia suprarrenal dos años antes con tratamiento regular, que ingresa por crisis suprarrenal y durante su hospitalización se solicitó una tomografía tóraco-abdominal donde se encontró bronquiectasias cilíndricas y de tracción asociadas a atelectasias cicatriciales en lóbulo pulmonar superior derecho, glándulas adrenales con aumento de volumen y focos de calcificación, así como una colección densa mediastinal posterior perivertebral que involucra además cuerpos vertebrales con focos líticos resortivos hallazgos compatibles con tuberculosis sistémica. Por este diagnóstico se inició tratamiento antituberculoso. En su evolución completó la primera fase con cuatro drogas y luego pasó a una segunda fase con isoniacida y rifampicina diaria con respuesta clínica favorable.

## INTRODUCCIÓN

La insuficiencia suprarrenal (IS) primaria también conocida como Enfermedad de Addison (EA), es consecuencia de una enfermedad intrínseca a la corteza adrenal, esta condición es resultante de una síntesis alterada de esteroides, destrucción suprarrenal o desarrollo anormal de la glándula que afectan la corteza suprarrenal ^1^.

En el grupo pediátrico, la etiología varía con la edad. En los neonatos y lactantes la causa más importante es la congénita, como la hiperplasia suprarrenal congénita por deficiencia de la enzima 21 hidroxilasa. En los niños más grandes puede ser por causas adquiridas como, por ejemplo, causas autoinmunitarias, procesos infiltrativos o infecciones. En este último caso, encontramos la tuberculosis ^2^.

La distribución de causas específicas de IS primaria varía según las poblaciones estudiadas ^3^^-^^5^. En general, las causas más frecuentes en niños varones de países desarrollados son las de etiología autoinmunitaria y la adrenoleucodistrofia, que constituye el 80-90 % de todos los casos ^6^^-^^8^; mientras que, en países en vías de desarrollo, la tuberculosis es aún una de las principales causas ^9^^,^^10^.

Se presenta el caso de un paciente con diagnóstico de IS primaria que ingresa por crisis suprarrenal y durante su evaluación se diagnostica una tuberculosis sistémica.

## REPORTE DE CASO

Paciente varón de 10 años con antecedente de diagnóstico de insuficiencia suprarrenal dos años antes de su ingreso. Es controlado de manera ambulatoria y con tratamiento regular con hidrocortisona y fludrocortisona. Dos semanas antes de su ingreso presenta dolor abdominal tipo cólico, difuso, intensidad moderada, asociado a sensación de alza térmica no cuantificada (de frecuencia interdiaria); una semana antes se agrega dolor en región dorsal y sudoración profusa a predominio nocturno, y tres días antes de su ingreso, se incrementan los síntomas presentando además náuseas y debilidad para deambulación, por lo que es traído a Emergencia. Al examen físico presentaba frecuencia cardiaca 107 latidos/minuto, frecuencia respiratoria 24 respiraciones/minuto, temperatura 36,7 °C, peso 23 kilos, talla 137 cm, presión arterial 79/54 mmHg (menor al percentil 5 para la edad y talla), IMC 12,25 (menor al percentil 1), llenado capilar menor de 2 segundos, ventilando espontáneamente, con signos de deshidratación. Se evidenció además hiperpigmentación cutánea a predominio de dedos de manos y pies, uñas (Figura 1A) y genitales, mucosas con manchas hipercrómicas en lengua y encías (Figura 1B, 1C). También, abdomen blando, depresible, doloroso a la palpación profunda de forma difusa, no visceromegalia. El resto del examen físico sin alteraciones.

**Figura 1:** Hiperpigmentación de la piel y mucosas

Al ingreso se encuentra hemoglobina 11,6 g/dL, leucocitos 12 260/uL, plaquetas 660 000/uL, glucosa 110 mg/dL, PCR 17,69 mg/dL, urea 71 mg/dL, creatinina 1,28 mg/dL, sodio 129 mmol/L, potasio 5,2 mmol/L, cloro 96 mmol/L. Gasometría: pH 7,36, pO2 46, pCO2 26,4, HCO3 15,1. Asimismo, cortisol (AM) 5,9 ug/dL, ACTH 50,2 pg/mL, TSH 7,18 uUI/ml, T4 libre 20,1 pmol/L; anticuerpo anti-tiroglobulina y anti-peroxidasa tiroidea negativos. Perfil de coagulación: tiempo de protrombina (TP) 14,8 segundos, INR 1,26, tiempo parcial de tromboplastina activada (TTPA) 37,4 segundos, tiempo de trombina (TT) 15,7 segundos, fibrinógeno 702 mg/dL y examen de orina: pH 6,0, densidad 1020, aspecto transparente, cuerpos cetónicos 2+, glucosa y proteínas (-), leucocitos 1-2/campo, hematíes 1-2/campo.

Es diagnosticado como una crisis adrenal y síndrome febril, indicándosele bolo de ClNa 0,9% 20 ml/kg en dos oportunidades e hidrocortisona 100 mg/m2 intravenosa. Posteriormente quedó con hidratación con solución glucosalina isotónica sin potasio a un volumen según método calórico. BK y Gene-Xpert en aspirado gástrico negativos. La radiografía de tórax mostró atelectasia en tercio superior de hemitórax derecho. La ecografía abdominal reveló glándulas suprarrenales de volumen incrementado con múltiples calcificaciones, mientras que en íleon terminal impresionaba paredes engrosadas, con pliegues marcados y engrosados. La tomografía tóraco-abdominal mostró en pulmones bronquiectasias cilíndricas y de tracción asociadas a atelectasias cicatriciales con finos infiltrados micronodulares en segmento posterior S3 de lóbulo pulmonar superior derecho (Figura 2), así como linfadenopatias mediastinales, paratraqueal derecha, infracarinal de hasta 15x12 mm y algunas axilares. Además, se evidenció colección densa mediastinal posterior perivertebral en mayor parte del eje dorsal con extensión subligamentaria anterior (Figura 3, flechas rojas), que involucraba además cuerpos vertebrales con focos líticos resortivos y cambios osteopénicos desde segunda hasta novena vertebra torácica (Figura 3, flechas azules). Ambas glándulas adrenales presentaban leve aumento de volumen, conteniendo gruesos focos de calcificación (Figura 4), y el bazo presentaba foco de calcificación puntiforme.

**Figura 2:** Bronquiectasias cilíndricas en segmento S3 en tomografía de tórax sin contraste.

**Figura 3:** Focos líticos resortivos desde T2 hasta T9 (flecha azul) y colección densa mediastinal perivertebral (flecha roja) en tomografía toraco-abdominal sin contraste.

**Figura 4:** Calcificaciones suprarrenales bilaterales en tomografía abdo-minal sin contraste.

Se diagnosticó como tuberculosis multisistémica con compromiso pulmonar, vertebral, suprarrenal y esplénico, y se inició tratamiento antituberculoso con esquema sensible de isoniacida 10 mg/kg/día, rifampicina 15 mg/kg/día, etambutol 20 mg/kg/día y pirazinamida a 30 mg/kg/día durante cuatro meses de primera fase y pasó a segunda fase con isoniacida y rifampicina diaria hasta la actualidad. Por parte de endocrinología salió de alta con hidrocortisona 1 mg/kg/día y fludrocortisona 0,1 mg/día con evolución favorable (Figura 5).

**Figura 5:** Línea de tiempo del cuadro clínico y evolución.

## DISCUSIÓN

Cuando se describió por primera vez el fallo adrenal crónico, alrededor del 50% de los casos fueron secundarios a tuberculosis. El *Mycobacterium tuberculosis* produce invasión y destrucción de la corteza suprarrenal, produciendo granulomas y necrosis, con posterior fibrosis y calcificación, llevando a una pérdida de función adrenal que puede ser irreversible ^11^. En reportes posteriores se muestra una disminución en la frecuencia de adrenalitis tuberculosa como causa de EA, sin embargo, en países donde la tuberculosis es endémica, o en la población inmunodeprimida, esta disminución no es tan marcada, con una incidencia variable entre 4,7 y 8,3 por millón ^11^^,^^12^. La tuberculosis puede comprometer ambas glándulas suprarrenales de forma primaria o puede ser parte de una enfermedad diseminada, como en el presente caso. En la literatura reportada se describe que el compromiso único de la glándula suprarrenal no es raro, pero, en su mayoría se encuentra compromiso de otros órganos ^13^.

Los signos clínicos de la EA se basan en la deficiencia de gluco y mineralocorticoides. Los signos debidos a la deficiencia de glucocorticoides son debilidad, anorexia y pérdida de peso. La deficiencia de mineralocorticoides contribuye a la hiponatremia, hiperkalemia, acidosis, taquicardia, e hipotensión ^1^. En el paciente la hiponatremia y la acidosis son evidente al momento del diagnóstico, así como la clínica de hipotensión que requirió tratamiento con bolos de ClNa 0,9%. La falta de retroalimentación negativa de glucocorticoides es responsable de los niveles elevados de ACTH presentes en el momento del diagnóstico inicial de IS, observándose valores aun ligeramente elevados en el cuadro actual, a pesar de que no suspendió su tratamiento en ningún momento. Los niveles elevados de ACTH y otros péptidos de la propia melanocortina (POMC), incluidas las diversas formas de hormona estimulante de melanocitos (MSH), provocan hipersecreción de melanina, estimulando la hiperpigmentación mucosa y cutánea ^1^, que persiste en el paciente y se intensifica durante la crisis. La hiperpigmentación suele ser un signo específico de IS y aparece cuando hay un compromiso de la glándula suprarrenal mayor al 90%, como consecuencia del efecto del incremento de la ACTH y MSH ^14^.

La crisis suprarrenal aguda es una afección potencialmente mortal en todas las edades. Los pacientes presentan malestar profundo, fatiga, náuseas, vómitos, dolor abdominal o en el costado, dolores o calambres musculares y deshidratación, que provocan hipotensión, shock y acidosis metabólica ^1^. En el paciente se encontró un shock hipovolémico con retención nitrogenada, debida a una injuria pre renal secundaria a la deshidratación. Esta condición requiere tratamiento inmediato, el cual se basa en una rápida reposición de líquidos para corregir la hipovolemia, el desequilibrio electrolítico y la hipoglucemia, así como la administración de hidrocortisona endovenosa ^1^^,^^2^, tal como se describe en el presente caso.

La descripción clínica al ingreso del paciente es típica de una crisis adrenal, a pesar de haber recibido tratamiento de reemplazo de manera regular. Esto nos hace resaltar la importancia de determinar la etiología del cuadro de insuficiencia suprarrenal para un adecuado control. Por el cuadro actual de fiebre sin foco evidente se solicitó una radiografía de tórax y posteriormente una tomografía tóraco-abdominal donde se evidenció el compromiso pulmonar, así como la afectación de las vértebras torácicas, las glándulas suprarrenales y el bazo.

El hallazgo de glándula suprarrenal con aumento de volumen se describe en múltiples reportes de adrenalitis tuberculosa ^15^, especialmente cuando el curso de la enfermedad es alrededor de 3 años ^16^. En este caso es evidente las calcificaciones bilaterales en la tomografía, lo que suele observarse conforme más tiempo pasa de la infección tuberculosa. Estos hallazgos, asociados a un compromiso vertebral asintomático, concuerdan con el curso crónico de la enfermedad.

Debido a que en la adrenalitis tuberculosa hay una destrucción mayor del 80% antes del inicio de síntomas, el tratamiento antituberculoso no siempre produce una recuperación completa de la función adrenal ^13^, sin embargo, se describe un mejor control adrenal luego del inicio del tratamiento. En el presente caso se inició con un esquema sensible que incluía isoniacida, rifampicina, etambutol y pirazinamida y se continuó el tratamiento con hidrocortisona y fludrocortisona.

En países como el Perú, con una alta prevalencia de tuberculosis, la adrenalitis tuberculosa siempre debe considerarse en el diagnóstico diferencial en pacientes escolares y adolescentes que presentan IS primaria.
